# Comparison of device-based physical activity and sedentary behaviour following percutaneous coronary intervention in a cohort from Sweden and Australia: a harmonised, exploratory study

**DOI:** 10.1186/s13102-020-00164-1

**Published:** 2020-05-09

**Authors:** Nicole Freene, Sabina Borg, Margaret McManus, Tarryn Mair, Ren Tan, Rachel Davey, Birgitta Öberg, Maria Bäck

**Affiliations:** 1 Physiotherapy, Faculty of Health, University of Canberra, Bruce, ACT 2617 Australia; 2 Health Research Institute, University of Canberra, Bruce, ACT Australia; 3 Department of Health, Medicine and Caring Sciences, Unit of Physiotherapy, Linköping University, Linköping, Sweden; 4 Department of Cardiology and Department of Health, Medicine and Caring Sciences, Linköping University, Linköping, Sweden; 5 Cardiology, Canberra Health Services, Garran, ACT Australia; 6 Exercise Physiology, Canberra Health Services, Garran, ACT Australia; 7 Centre for Research and Action in Public Health, University of Canberra, Bruce, ACT Australia; 8 Department of Occupational Therapy and Physiotherapy, Sahlgrenska University Hospital, Gothenburg, Sweden

**Keywords:** Acute coronary syndrome, Secondary prevention

## Abstract

**Background:**

Few studies have measured device-based physical activity and sedentary behaviour following a percutaneous coronary intervention (PCI), with no studies comparing these behaviours between countries using the same methods. The aim of the study was to compare device-based physical activity and sedentary behaviour, using a harmonised approach, following a PCI on-entry into centre-based cardiac rehabilitation in two countries.

**Methods:**

A cross-sectional study was conducted at two outpatient cardiac rehabilitation centres in Australia and Sweden. Participants were adults following a PCI and commencing cardiac rehabilitation (Australia *n* = 50, Sweden *n* = 133). Prior to discharge from hospital, Australian participants received brief physical activity advice (< 5 mins), while Swedish participants received physical activity counselling for 30 min. A triaxial accelerometer (Actigraph GT3X/ActiSleep) was used to objectively assess physical activity (light (LPA), moderate-to-vigorous (MVPA)) and sedentary behaviour. Outcomes included daily minutes of physical activity and sedentary behaviour, and the proportion and distribution of time spent in each behaviour.

**Results:**

There was no difference in age, gender or relationship status between countries. Swedish (S) participants commenced cardiac rehabilitation later than Australian (A) participants (days post-PCI A 16 vs S 22, *p* < 0.001). Proportionally, Swedish participants were significantly more physically active and less sedentary than Australian participants (LPA A 27% vs S 30%, *p* < 0.05; MVPA A 5% vs S 7%, *p* < 0.01; sedentary behaviour A 68% vs S 63%, *p* < 0.001). When adjusting for wear-time, Australian participants were doing less MVPA minutes (A 42 vs S 64, *p* < 0.001) and more sedentary behaviour minutes (A 573 vs S 571, *p* < 0.001) per day. Both Swedish and Australian participants spent a large part of the day sedentary, accumulating 9.5 h per day in sedentary behaviour.

**Conclusion:**

Swedish PCI participants when commencing cardiac rehabilitation are more physically active than Australian participants. Potential explanatory factors are differences in post-PCI in-hospital physical activity education between countries and pre-existing physical activity levels. Despite this, sedentary behaviour is high in both countries. Internationally, interventions to address sedentary behaviour are indicated post-PCI, in both the acute setting and cardiac rehabilitation, in addition to traditional physical activity and cardiac rehabilitation recommendations.

**Trial registrations:**

Australia: Australian New Zealand Clinical Trials Registry (ANZCTR): ACTRN12615000995572. Registered 22 September 2015, Sweden: World Health Organization Trial Registration Data Set: NCT02895451.

## Background

Percutaneous coronary intervention (PCI) is the most commonly used procedure for myocardial revascularisation globally [1, 2]. Both Australian [3–5] and European (applied in Sweden) clinical guidelines [6] for the management of acute coronary syndrome (ACS) recommend that all individuals hospitalised with ACS, including those undergoing a PCI, should receive physical activity counselling and referral to a cardiac rehabilitation program prior to discharge. Meta-analysis clearly confirm that participation in exercise-based cardiac rehabilitation is associated with positive health benefits in terms of reduced cardiac mortality, risk of hospital readmission and improved aerobic capacity for patients with an ACS [7]. Additionally, studies assessing physical activity in patients with ACS have established an inverse relationship between increased levels of physical activity and mortality [8–10]. Although, these studies are limited to self-reported physical activity, entailing a risk of over- or under-estimating physical activity, as well as issues of recall and response bias [11]. Accelerometry is considered a superior method of physical activity measurement, with lower levels of variability observed for validity and reliability, overcoming limitations of self-reports [12]. However in patients with ACS, few studies have measured device-based physical activity and sedentary behaviour (a risk factor for all-cause mortality in people with cardiovascular disease [13]) following a PCI [14–17]. All of these studies have used different measurement devices (Sensewear Armband, GENEActiv, ActiCal, ActiGraph) and different measures of physical activity and sedentary behaviour (minutes per day, percentage wear time, ≥ 1 day exercising) making it difficult to compare results. No studies have used the same methods to compare device-based physical activity and sedentary behaviour between countries in this population.

International comparisons provide a broader perspective of health and health care, potentially identifying best practice and factors that may influence outcomes [18]. The data (definitions, participants, time period) and methods used in these comparisons should be similar so differences, if any, can be clearly identified and appropriate conclusions can be made [18]. Australia and Sweden both have a very high Human Development Index, a composite measure of life expectancy, education and income, ranked third and seventh in the world respectively [19]. They are both members of the Organisation for Economic Co-operation and Development, having similar levels of economic development [20]. Coronary heart disease is the leading cause of death and disease burden in Australia [21] and in Sweden [22]. In 2000, 21,784 PCI procedures were performed in Australia, increasing to 41,200 in 2016 [2, 23]. Approximately 75% of Australians with a diagnosis of ST-elevated myocardial infarction were treated with a PCI procedure in 2012–2015 [24]. In Sweden, 10,000 PCI procedures were performed in 2000, which has doubled to over 20,000 procedures in 2018 [25]. Therefore, it appears it is appropriate to quantify and compare physical activity and sedentary behaviour in this group of patients following a PCI in these two countries.

In cardiac rehabilitation (including PCI participants), device-based physical activity levels have been reported as low (approximately 11 min moderate-intensity physical activity a day [26, 27]), and sedentary behaviour high (approximately 8–10 h a day [17, 27]). Currently, it is difficult to compare device-based physical activity in cardiac populations around the world, with a variety of different data collection and processing methods utilised. Here we describe a method to assess physical activity and sedentary behaviour in post-PCI participants’ on-entry into cardiac rehabilitation. This will allow comparison of physical activity and sedentary behaviour levels internationally post-PCI, providing an indication of differences between countries and the discussion of possible explanatory factors. Results could guide acute post-PCI management and cardiac rehabilitation guidelines internationally. The aim of the study was to compare device-based physical activity and sedentary behaviour, using the same methods, following a PCI on-entry into centre-based cardiac rehabilitation in two countries.

## Methods

A cross-sectional study was conducted at the commencement of phase II cardiac rehabilitation at one centre in Australia and Sweden. In Australia, the cardiac rehabilitation program involved both exercise and education sessions at every attendance. In Sweden, patients were offered exercise-based cardiac rehabilitation and education sessions separately. Most patients start the weekly education sessions 1 week from hospital discharge and before they commence the exercise sessions. The education sessions cover three different topics: 1) Heart disease and risk factors, 2) Psychological aspects related to heart disease, 3) Diet and exercise. The majority of patients do not complete the education session on diet and exercise until after they start the exercise sessions. In this study, Swedish participants were included prior to the start of the exercise sessions. As all participants were assessed on-entry into exercise-based cardiac rehabilitation, any differences in exercise-based cardiac rehabilitation guidelines between countries were not relevant as no participants had commenced the exercise sessions. The participants were a subset of participants from larger studies conducted in both countries. The Australian and Swedish study protocols have been described elsewhere, as well as results from the larger Australian cohort study [28–31].

### Participants

Eligible participants were aged ≥18 years (Australia and Sweden) and < 75 years (Sweden) and had agreed to start the cardiac rehabilitation program. Consecutive participants were included if they had stable coronary heart disease and had received a PCI +/− myocardial infarction. Participants were recruited between November 2015 and August 2016 in Australia, and between January 2016 and August 2018 in Sweden. All participants provided written consent.

### Post-PCI physical activity and cardiac rehabilitation advice

At the Australian hospital, all post-PCI patients are seen by cardiac rehabilitation or cardiology nurses prior to discharge and are encouraged to start regular, low-to-moderate intensity physical activity, starting slowly and progressing gradually. This advice is brief, approximately 5-min, and is supported with written material from the National Heart Foundation, initially encouraging 5 to 10 min strolls twice a day [32]. At the Swedish hospital, patient’s post-PCI meet with a physiotherapist for 20 to 30-min prior to discharge for physical activity counselling [33]. Patients post-PCI are encouraged to start regular moderate-to-vigorous physical activity (MVPA) as soon as possible, and the recommended dose is consistent with the general global recommendation on physical activity for health; 30 min of at least moderate intensity aerobic activity on 5 days a week [6, 34]. In Australia and Sweden, all patients are referred and encouraged to attend an outpatient cardiac rehabilitation program, commencing soon after discharge from hospital.

### Outcome measures

#### Physical activity and sedentary behaviour

A triaxial commercial accelerometer (ActiGraph ActiSleep or GT3X^1^) was used to objectively assess physical activity and sedentary behavior in both countries. Participants were asked to wear the monitor on their right hip for 24-h per day (Australian sample) or during waking hours (Swedish sample), for 7-consecutive days. For the Australian data, to eliminate sleep time a time filter was applied from 7 am to 10.30 pm, the average time out-of- and into-bed each day based on participants’ surveys, only analyzing data between these times. Participants were instructed not to wear the accelerometer in water. The triaxial accelerometer captures movement around three axes; vertical (y-axis), horizontal (x-axis) and perpendicular (z-axis). Vector magnitude is a composite measure of all 3 axes (√x^2^ + y^2^ + z^2^). A review of the literature was conducted to determine the most suitable parameters for accelerometer data processing in participants with coronary heart disease [17, 27, 35–37].

#### Accelerometer sampling, epoch length and wear time

All data was sampled and downloaded as raw data (30 Hz), converted to 15-s epochs (time interval), and then counts per minute (cpm) using the Actilife^1^ software [17, 27]. A ‘count’ is the unit of measure for activity for ActiGraph’s activity monitors [38]. Data was screened, excluding data if: < 10 h per day wear time (non-wear defined as > 60 consecutive minutes where there is zero activity, with no allowance of epochs with counts above zero) and less than 4 days of valid data [17, 27, 35]. If there was more than 7 days of valid data, all valid days were used to calculate the average [17].

#### Accelerometer cut-points

The Sasaki vector magnitude 3 cut-points were used to determine time spent in MVPA (≥2690 cpm) [17, 27, 35, 36]. To measure sedentary behaviour, the vector magnitude cut-point was used (< 150 cpm), categorizing light physical activity (LPA) as 150–2689 cpm [17, 27, 35, 37]. These cut-points have not been validated in coronary heart disease participants, although they have been used in prior research in this population [17, 27]. Both of these cut-points have been validated in younger, generally healthy participants [36, 37]. Estimating daily time spent in physical activity and sedentary behavior was calculated by dividing the total time spent (minutes) in each threshold by the number of valid days. In addition, daily time spent in LPA, MVPA and sedentary behavior was expressed as percentage of total daily wear time.

#### Distribution of physical activity and sedentary behaviour

MVPA bout data used a minimum bout length of 10 min, allowing for 2 min of counts less than the MVPA threshold within this time [17, 27, 35, 36]. Daily time in MVPA bouts was calculated by dividing total time in MVPA bouts by the number of valid days. Sedentary behaviour bout data used a minimum length of 10 min, with no drop time [27]. Sedentary bouts are the number of bouts (≥ 10 consecutive minutes) of sedentary time per day. Average sedentary bout length is the total time in sedentary bouts divided by the total number of bouts. A break is an interruption in sedentary time (≥150 cpm). In the data analysis ‘ignore first sedentary break of each day’ was used to remove sedentary time accrued while the device was removed at night [27].

#### Secondary outcomes

Secondary outcomes included body mass index (BMI, kg/m^2^), resting blood pressure, and anxiety and depression (Hospital Anxiety and Depression Scale, HADS [39]). The HADS questionnaire is a 14-item self-report questionnaire comprised of 4-point Likert-scaled items covering the occurrence of symptoms of anxiety (HADS-A) and depression (HADS-D) over the past 2 weeks. Each item on the questionnaire is scored from 0 to 3, so that a person can score between 0 (best outcome) and 21 (worst outcome) for either anxiety or depression. The normal range is considered 0–7 on each sub-scale. Sociodemographic and other clinical information, including time since PCI, were also collected.

### Statistical analysis

Descriptive analyses were completed. Normality of the data was assessed using the Kolmogorov-Smirnov test. For parametric data, unpaired t-tests were used, and for the accelerometer data, ANCOVAs were used controlling for accelerometer wear time. For non-parametric data, independent samples Mann–Whitney U test with a 95% confidence interval were used to assess differences between countries. Chi-square analyses were performed to determine if there were significant differences in distribution of categorical data between countries. All analyses were conducted using SPSS^2^ version 25. Significance level was set at *p* < 0.05.

## Results

There was no difference in age, gender or relationship status of participants between countries (Table 1). Swedish participants had a higher level of education and started cardiac rehabilitation later. One third of Australian participants were born in other countries, compared with < 1% of Swedish participants. Australian participants had a higher BMI, a higher proportion of participants with type 2 diabetes and a lower resting DBP. Additionally, Australian participants had higher levels of depression, although both countries had low levels of depression overall.

**Table 1:** Participant characteristics

Characteristic	Australia (*n* = 50)	Sweden (*n* = 133)
Age (years), mean (SD)	62.8 (9.2)	62.1 (8)
Gender, n male (%)	41 (82)	110 (83)
Country born, n (%)		
Australia	30 (67)	–
Sweden	–	128 (99)
Other	15 (33)	1 (1)
Relationship status, n partner (%)	33 (75)	103 (77)
Education level, n tertiary (%)	28 (64)	123 (93)***
Current smoker, n no (%)	44 (98)	123 (93)
Type 2 diabetes, n no (%)	35 (78)	121 (91)*
Days post-PCI	16 (13–23.3)	22 (17–28)***
Measures of disease risk		
Body mass index (kg/m^2^)	28 (25.9–31.9)	26.2 (24.7–29.3)**
Systolic blood pressure (mmHg)	121 (115–135)	126 (115–137)
Diastolic blood pressure (mmHg)	70 (65–76.5)	80 (76–88)***
HADS-Anxiety	3 (1–6)	3 (1–6)
HADS-Depression	3 (1–4)	1 (1–3)**
HADS-total	5 (3–10)	5 (2–8)

Data presented as median (IQR) unless otherwise specified. *P ≤ 0.05 **P ≤ 0.01 ***P ≤ 0.001. PCI; percutaneous coronary intervention; *HADS* Hospital anxiety and depression scale

**Fig. 1:**
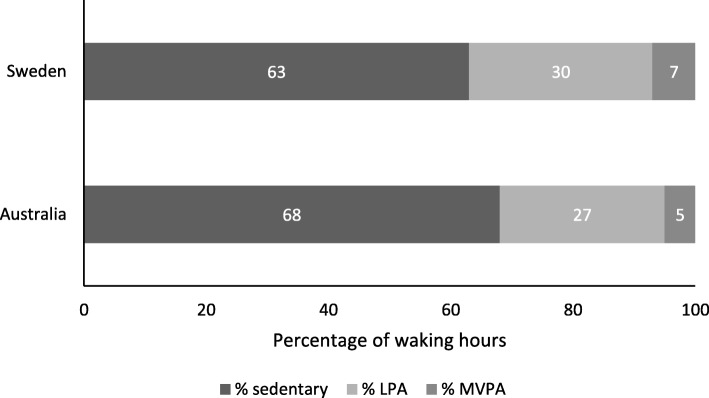
Percentage of waking hours spent in sedentary behaviour, light (LPA) and moderate-to-vigorous physical activity (MVPA) at baseline

Swedish participants spent a greater proportion of the day in MVPA (*p* = 0.0001) and LPA (*p* = 0.045) compared to Australian participants (Fig. 1, Table 2). Swedish participants also spent a significantly smaller proportion of their day in sedentary behaviour (*p* = 0.001, Fig. 1, Table 2), and completed less sedentary bouts and breaks (Table 2) compared to Australian participants. Between countries, there was no significant difference in the duration of sedentary bouts, with the mean sedentary bout approximately 20 min in length (Table 2). After adjusting for wear time, Swedish participants completed significantly more steps per day compared to Australian participants (Table 2).

**Table 2:** Physical activity and sedentary behaviour characteristics

Actigraph 15 s	Australia	Sweden	*P*-value
MVPA mins/day	42.07 (26.85)	63.78 (29.65)	0.0003
MVPA bout mins/day, median (IQR)	3.27 (0–18.01)	17.4 (6.04–32.27)	0.002
LPA mins/day	232.95 (66.04)	268.2 (62.49)	0.046
Sedentary mins/day	572.77 (80.6)	570.71 (90.39)	0.001
Duration sedentary bouts/day (mins)	20.27 (2.99)	19.38 (2.65)	0.089
Number of sedentary bouts/day	12.91 (4.08)	11.65 (4.36)	0.004
Number sedentary breaks/day	11.91 (4.08)	10.65 (4.36)	0.004
Wear time mins/day	847.79 (58.90)	902.72 (81.44)	0.00002
VM counts/day	372,023 (136503)	493,717 (153313)	0.0002
Steps/day	6115 (2257)	8122 (3010)	0.0004

Data presented as mean (standard deviation) unless otherwise specified. *MVPA* Moderate-to-vigorous physical activity, *LPA* Light physical activity, *VM* Vector magnitude

## Discussion

Following a PCI, Swedish participants were more active and less sedentary than Australian participants commencing cardiac rehabilitation. Although, the minimal difference (approximately 2 min) in sedentary behaviour between both countries is unlikely to be clinically significant. Rogerson et al. (2016) examined the relationship between sedentary behaviour (television viewing time) and all-cause mortality in participants with CVD, and found that the unadjusted mortality rate increased for every one-hour increment in television viewing time [40]. Regardless of this difference, participants in both countries spent a large proportion of their day sitting or lying while awake (63–68%), with approximately 9.5 h per day spent in sedentary behaviour.

A recent meta-analysis of 1 million participants found that too much sedentary behaviour may be above 9-h of device-based sedentary time, with increased mortality risk above this daily amount [41]. Reducing sedentary behaviour has recently been added to the public health physical activity guidelines in Australia and America [42, 43]. Yet, no guidelines for ACS management, or cardiac rehabilitation, appear to include recommendations to reduce sitting time. There is evidence that all-cause mortality decreases with reductions in sedentary behaviour in people with CVD, even when no MVPA is completed [13, 40]. Therefore, decreasing sedentary behaviour may be crucial in ACS patients to prevent recurrent cardiac events, particularly when there is some evidence that nearly half of ACS patients (84% post-PCI) do not exercise at all during weeks 2–5 post-discharge when measured using accelerometry [15], and our results indicate that post-PCI participants are sitting or lying on average 9.5 h per day within 2–3 weeks of their procedure.

The provision of physical activity advice, including referral to cardiac rehabilitation, to individuals with ACS is recommended in Australia and Sweden prior to hospital discharge [3, 5, 32, 33]. Although, the guidelines in Australia are not clear how much physical activity advice should be provided, whether it should include written advice and who should provide the lifestyle counselling. In contrast, Swedish physiotherapists provide physical activity and exercise advice before hospital discharge for approximately 20 to 30-min. Post-PCI patients are recommended to initiate MVPA (versus low-to-moderate physical activity in Australia) as soon as possible after discharge from hospital. It is recognised that it may be challenging to provide physical activity advice prior to discharge, when the average length-of-stay in hospital following a PCI has been reported as 1–2 days [2, 44]. To investigate whether ACS inpatients were receiving lifestyle advice, a large prospective audit of 2299 ACS inpatients in Australian and New Zealand public and private hospitals was conducted in 2012 [45]. This snapshot audit found that only 43% of ACS patients received physical activity advice prior to discharge in these countries. In Sweden, a recent study found that in 78% of the centres surveyed (*n* = 78), all ACS patients met with a physiotherapist for physical activity and exercise training counselling before discharge [33]. However, only 27% of centers provide written personalized information on lifestyle goals [33]. The difference in education provided between counties prior to discharge may be an explanatory factor for increased levels of physical activity within the Swedish participants at the commencement of cardiac rehabilitation. Further investigation is required of post-PCI physical activity advice prior to discharge to determine who should deliver this information, how much information should be provided and how this information should be delivered, for example, supported by written material. This may have an impact on the patient’s confidence to move upon discharge.

Other possible explanatory factors for the differences in physical activity levels are a lower level of education and a higher proportion of participants born in other countries in the Australian sample, which may have contributed to a lower level of health literacy [46]. Swedish participants also commenced cardiac rehabilitation approximately 6 days later than Australian participants, which may have increased their confidence to move. The statistically significant differences in BMI and diastolic blood pressure may not be clinically significant as both Australian and Swedish participants were overweight, with normal range diastolic blood pressures.

Self-report data suggests that a higher proportion of the Swedish adult population are meeting the physical activity guidelines, that is, 150 min of MVPA per week [34], compared to the Australian adult population [21, 47]. Sixty-seven percent of Swedish adults 18–64 years are sufficiently active compared with 48% of Australians in the same age group. This difference becomes larger when comparing older adults (≥65 years old), with 55% of Swedish older adults meeting the physical activity guidelines compared with 25% of Australian older adults. There are recognised limitations with self-report physical activity data, including over-reporting and differences between surveys [11] but this indicates that Swedish post-PCI participants may have had a higher level of physical activity pre-PCI than the Australian participants and this may partly explain the differences. These differences in pre-existing physical activity levels may be due to a number of factors including the built environment, active transport systems, physical activity promotion in schools, the workplace and the health sector [47, 48]. Some of these factors may also have contributed to an increased physical activity level post-PCI. Despite the differences in general public self-reported physical activity levels, device-based sedentary time in a large sample of Australian (*n* = 698, mean (SD) age 57.9 (9.9) years) and Swedish adults (*n* = 851, 56% women, mean (SD) age 66.7 (10.2) years) appears similar (sedentary behaviour A 8.8 vs S 8.2 h per day) [49, 50]. Further investigation of factors that influence patients’ physical activity and sedentary behaviour internationally following a PCI is indicated to contribute to the development of clinical guidelines and the improvement of services. A broader systems approach to physical activity for cardiac health may be indicated, with successful primary prevention strategies potentially contributing to higher baseline physical activity levels and a more readily acceptable need to maintain or increase physical activity levels for secondary prevention of ACS.

### Study limitations

This was a small cross-sectional study, providing a snapshot of post-PCI physical activity and sedentary behaviour levels from only one centre in each country, over a similar time frame. There were also less participants in the Australian sample compared to the Swedish sample, which may have led to less conclusive results for this cohort. Generalizability of the results internationally is limited, as the participants were predominantly males, in a relationship and on average, 62 years old. There are limited data on sick leave and whether patients in the two cohorts had returned to work after their PCI. This could have affected the physical activity and sedentary behaviour levels, however, the time for sick leave are similar between countries (1 week). These participants were also potentially more motivated to adhere to lifestyle modifications as they had agreed to participate in cardiac rehabilitation. The Swedish participants may also have been more motivated as they had received some education sessions prior to starting the exercise sessions. The accelerometer cut-off thresholds used may also not have been appropriate for use in the cardiac population, inaccurately classifying physical activity and sedentary behaviour, with no validated methods available for cardiac participants. Additionally, the general time filter applied to the Australian data may have resulted in an over-estimation of sedentary time and decreased wear-time for some participants. Furthermore, the Actigraph monitor may not be the most appropriate monitor to measure sedentary behaviour, with the activPAL monitor considered a more precise monitor for measuring sedentary time [37]. Although, a major strength of this study is the use of the same accelerometers (ActiGraph) and using the same procedures for accelerometer data sampling, cleaning and analysis from post-PCI populations in two countries.

## Conclusion

Swedish post-PCI participants when commencing cardiac rehabilitation are more physically active than Australian participants in this study. Potential explanatory factors are differences in post-PCI physical activity education between countries and pre-existing physical activity levels. Despite this, sedentary behaviour is high in both countries. Interventions to address sedentary behaviour are indicated post-PCI, in both the acute setting and cardiac rehabilitation, in addition to traditional physical activity and exercise recommendations. Internationally, further investigation of factors that influence patients’ physical activity and sedentary behaviour following a PCI is indicated.

## Data Availability

The datasets used and/or analysed during the current study are available from the corresponding author on reasonable request.

## Abbreviations

A: Australia
ACS: Acute coronary syndrome
CVD: Cardiovascular disease
LPA: Light physical activity
MVPA: Moderate-to-vigorous physical activity
PCI: Percutaneous coronary intervention
S: Sweden

## Footnotes

1 ActiGraph, Fort Walton Beach, Florida

2 IBM, Armonk, New York
